# Unlocking the Potential: Millets and Their Impact on Diabetes Management

**DOI:** 10.7759/cureus.59283

**Published:** 2024-04-29

**Authors:** Ansar Ahamed V.P., Abhishek Joshi, Abhay Mudey, Sonali Choudhari, Juhi Raut, Sana Ahmed

**Affiliations:** 1 Community Medicine, Jawaharlal Nehru Medical College, Datta Meghe Institute of Higher Education and Research, Wardha, IND

**Keywords:** nutrition, millets, glycemic index, diabetes mellitus, diet

## Abstract

Many studies, including case studies, meta-analyses, and randomized trials, have demonstrated the benefits of a low-carb diet in the management of obesity, diabetes, and pre-diabetes. Numerous studies suggest that diets low in carbohydrates are safe and can greatly enhance the management of both forms of diabetes as well as the general health of those who have the disease. When used in conjunction with effective therapy, this diet can result in weight loss, decreased prescription dosages, and in certain cases, remission from type 2 diabetes. Globally, there has been a notable surge in the prevalence of diabetes cases as a result of factors such as population growth, aging, urbanization, rising obesity rates, and declining physical activity. Diabetes can be controlled in large part by diet, and millets having low-glycemic index (GI) have become more significant as they release glucose into the bloodstream at a very slow rate. Creating a low-glycemic meal mix with locally sourced ingredients is crucial for daily diet plans. Dietary changes, particularly the addition of millet, can help prevent and manage diabetes mellitus. Eating practices have long been acknowledged for their important role in promoting health and wellness through the consumption of nutrient-dense meals. The health benefits of millet, an underappreciated food crop, are numerous and include low GI, high-fiber content, non-acid-forming potential, polyunsaturated fatty acids (PUFAs), and gluten-free status. Apart from staple crops like wheat and rice, millets are also very healthy and useful, and they have an immense amount of opportunity to aid in the global fight against food insecurity, which is a problem that many countries now confront. Millets are high on the list of recommended foods because of their many health advantages and antioxidant characteristics. Diets that are low in carbohydrates, low in GI, Mediterranean, and very low in calories are now popular. Diabetes can be managed with a nutritious diet, regular exercise, cessation of smoking, and maintenance of a healthy body weight. Furthermore, calorie restriction, the use of low-GI meals, and an increase in fiber content are all possible nutritional strategies in the management of diabetes.

## Introduction and background

A healthy diet is one of the lifestyle-based therapies that are most important for treating diabetes, and many nations have set their own dietary guidelines regarding this. Every nation shares a commitment to providing personalized nutrition therapy based on individual preferences and treatment objectives. Unfortunately, due to national and even regional differences within a single country, as well as variations in body size, age, lifestyle, and cuisine, nutritional therapy cannot be standardized [1]. Nowadays, with technology being so easily available, people are leading highly sedentary, stressful lives devoid of adequate sleep and a well-balanced diet. Among the top 10 causes of death, along with cancer, respiratory diseases, and cardiovascular diseases, diabetes mellitus (DM) is one of the most crucial health issues of the twenty-first century [2]. The World Health Organization (WHO) reports that noncommunicable diseases (NCDs) accounted for 74% of all deaths worldwide in 2019 [3]. Diabetes was rated as the tenth greatest cause of mortality worldwide in 2019, with 1.9 million fatalities [3]. It is projected that 592 million individuals will lose their lives to diabetes by the year 2035 [4]. According to the WHO, low- and middle-income nations have the fastest-rising rates of diabetes prevalence [5].

India is now a hub for conditions like obesity, atherosclerosis, DM, hypertension (HTN), and heart disease. One of the global epicenters of the DM pandemic is India. The past 40 years have seen an exponential rise in the prevalence of DM in India due to rapid socioeconomic development, demographic shifts, and greater susceptibility for Indians [6]. A study by the Indian Council of Medical Research (ICMR) estimates that over 101 million Indians have diabetes, which is much higher than earlier reported figures [7]. A minimum of 136 million individuals, or 15.3% of the populace, suffer from prediabetes [7].

In poorly managed patients with DM, glucose levels remain constantly high, either due to an inefficient supply of insulin or as a result of genetic, acquired, or deficits in pancreatic hormone synthesis. Type 1 diabetes, commonly referred to as insulin-dependent diabetic mellitus (T1DM), makes up 5%-10% of all cases of diabetes. This autoimmune disease causes insulin deficiency and eventually hyperglycemia by attacking and killing pancreatic β-cells using T-cells. T2DM, commonly referred to as adult-onset diabetes or non-insulin-dependent diabetic mellitus, accounts for the majority of diabetes cases, approximately 90% to 95%. Insulin resistance and β-cell dysfunction are the two main insulin-related disorders that define this kind of diabetes [8]. It is becoming a serious public health issue in both industrialized and developing nations. Recent research indicates that diabetes is becoming more common among Indians residing in both rural and urban areas, with southern India experiencing the most increase in the past 20 years [9]. It is a lifestyle-related disorder that can be avoided and managed with modifications to diet and prescribed medications. For diabetics, a wide range of dietary recommendations and options are easily accessible. Certain individuals have even offered guidance regarding food categories right down to the kind of grains. A family of small-seeded, nutrient-dense food crops known as millets is widely grown on marginal soil in dry parts of the world [10].

As a nutritional choice, millets have been getting more attention recently for their potential to treat diabetes. Indeed, there is evidence to suggest that millets have a variety of benefits that make them a healthy dietary choice for those with diabetes [11]. Apart from the staple food crops that people have been eating for a long time, like rice and wheat, millet is still very beneficial and nutritious. It is an underutilized crop that has many health benefits, the most important of which are that it is gluten-free, low in glycemic index (GI), high in fiber, and contains polyunsaturated fatty acids (PUFAs) [12]. Millets are incredibly nutrient-dense food crops that are high in iron, calcium, magnesium, zinc, potassium, fatty acids, protein, and dietary fiber. They are also especially rich in vitamins, particularly the B complex [13]. Dietary fibers from millet serve as prebiotics, promoting the growth of a balanced gut microbiota [14]. Millets include dietary fiber, which can aid with blood glucose management, slow down the rate at which glucose is absorbed in the small intestine, and lower the food's GI [15]. Patients with celiac disease can frequently eat millet because they are a gluten-free cereal grain [16]. Higher amounts of phytochemicals such as phenolics, tannins, carotenoids, and flavonoids have strong antioxidant activity and help in slowing tumor growth [17]. Millets are also well known for their possible health advantages, such as cardiovascular diseases, DM, thyroid issues, celiac diseases, and HTN. Millets have a few distinctive qualities: in contrast to other major cereals, millet is noted for its short growing season, ability to endure droughts, and tolerance to illnesses and pests [18]. Including millets in meals along with staple foods and vegetables grown nearby offers significant health advantages due to the high fiber, macronutrient, micronutrient, vitamin, and mineral content that millets possess. They can also aid in the fight against chronic illnesses. Smallholder farmers typically cultivate millets, an indispensable food crop, throughout the semi-arid and dry regions of the world. Millet is known in popular culture as *Nutri cereals* because of the abundance of vital nutrients, including fiber, protein, and micronutrients, they contain. Millets are a key component in the fight against malnutrition and the advancement of food security.

The United Nations (UN) and the Food and Agriculture Organization have declared 2023 to be the *International Year of Millets* (IYM2023), highlighting the nutritional importance of these Poaceae family grains to raise public awareness of their nutritional value and health benefits [19]. The aim of this study is to convey the nutritional advantages of millet and its effectiveness in preventing, treating, and controlling DM.

## Review

Methodology

To find the articles, an automated search of PubMed, Google Scholar, and Web of Science databases was conducted and that may be included in this review. The search was limited to publications that were available until February 2024. We obtained 181,521 articles from the search engines using search terms like "diet," "diabetes mellitus," "glycemic index," and their synonyms. After filtering the results by full free-text article availability and articles from the year 1990 till February 2024, we obtained 67,522 articles. After screening the title and abstract, 78 articles were selected. Finally, after reading the full-text articles available, a total of 39 articles were used for this article. Only English-language literature was included in the search parameters.

Discussion

Millets and Their Significance

**Figure 1:**
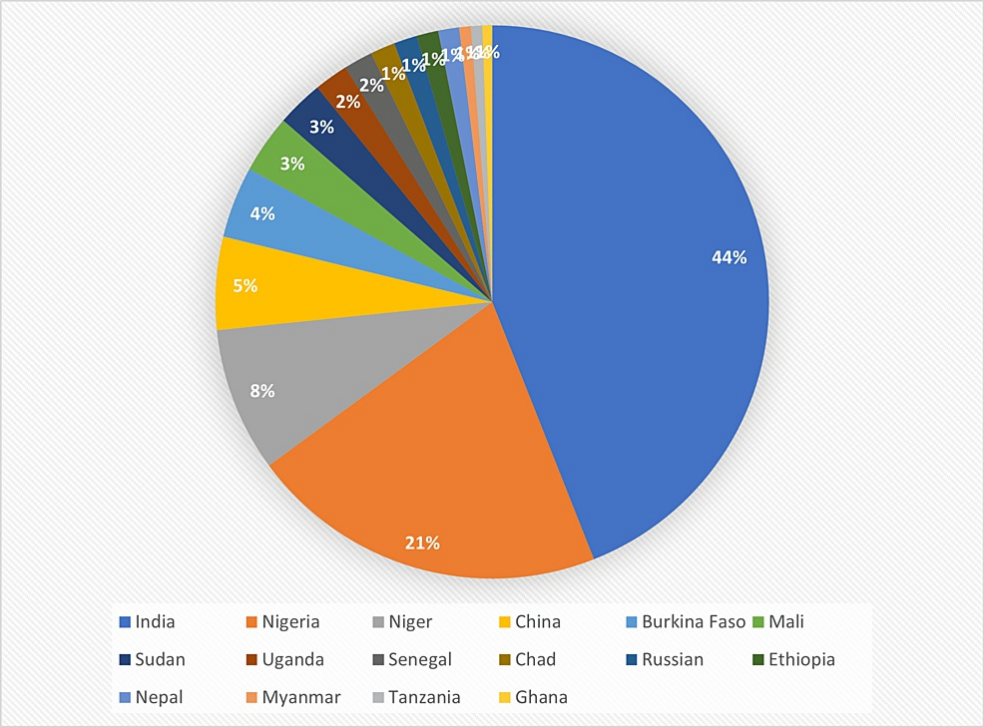
Millets: Global pattern of consumption. Source: [21]. Image credit: Ansar Ahamed VP.

A group of historically significant crops known as millet have bright prospects for reducing food insecurity. They are grown in the tropical semi-arid regions of Africa and Asia. Millets are grasses that belong to the Poaceae (Gramineae) family. In general, the name *millet* refers to a diverse collection of crops distinguished by their small, coarse grains. Depending on the size of their grains, these crops are divided into two groups: great/large millets and little millets [20]. Some of the minor millet kinds are finger millet (Eleusine coracana), proso millet (Panicum miliaceum), foxtail millet (Setaria italic), and kodo millet (Paspalum scrobiculatum). The great and large millets include pearl millet (Pennisetum glaucum) and sorghum (Sorghum bicolor) [20]. It is an easily cultivable crop that could be produced in drought-prone locations with lower-than-average annual precipitation because it is rain-fed and requires little irrigation. The amount of water required by millets is remarkably small [21]. Finger millet, pearl millet, and sorghum require less rainfall than banana and sugarcane and 30% less rainfall than rice. While all millets grow without irrigation, it takes 4,000 L of water to grow 1 kg of rice. This could prove to be extremely beneficial for the country, particularly in light of the coming decades-long climate disaster [21]. Figure 1 shows the global pattern of consumption of millets [21].

**Figure 2:**
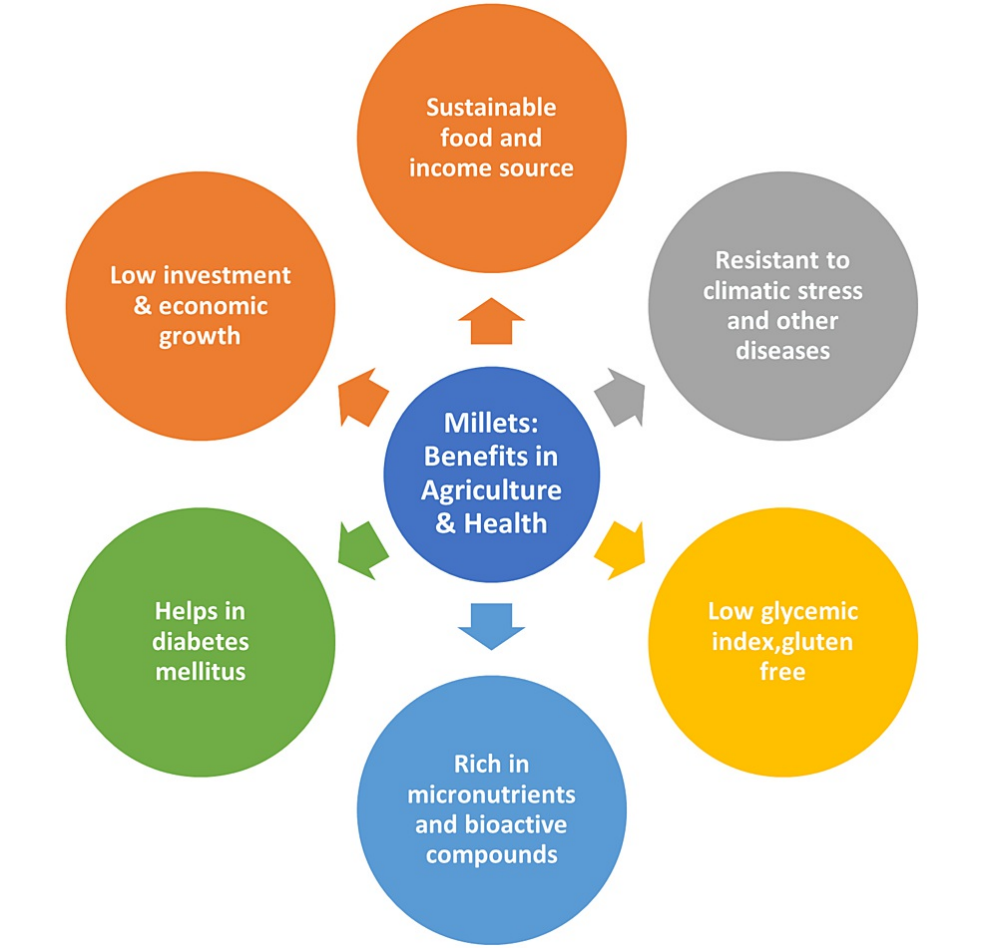
Health and agricultural benefits of millet. Image credit: Ansar Ahamed V.P.

Millets possess the capacity to become the food of security in the future when the world faces a food and water catastrophe. Numerous types of millet are consumed in Indian cuisine. The most widely used millets in India are finger millet or ragi, jowar, sorghum, and pearl millet or bajra [22]. Millets are the mainstay of Indian cuisine and have been for many generations because India leads the globe in millet production. About 40.20% of the global millet is produced annually in India [23]. Figure 2 shows the health and agricultural benefits of millets.

The consumption of nutrient-rich cereals is associated with several health benefits, including lowering blood sugar levels (diabetes), controlling blood pressure, and providing protection against thyroid, cardiovascular, and cancer diseases [12]. Farmers have economic prospects due to the increased demand for millets, particularly in areas where they are historically farmed. Along the value chain, there is also potential for value addition through the processing and sale of products based on millet, which would provide money and jobs. There is an increasing trend in popular diets to include traditional and alternative grains, such as millet, and to diversify food options. Along the millet value chain, this movement offers chances for value addition and market expansion. To adapt to shifting customer demands, entrepreneurs and food corporations might invest in creating novel millet-based products, including baked goods, and cereals for breakfast and snacks.

GI and Millets

**Figure 3:**
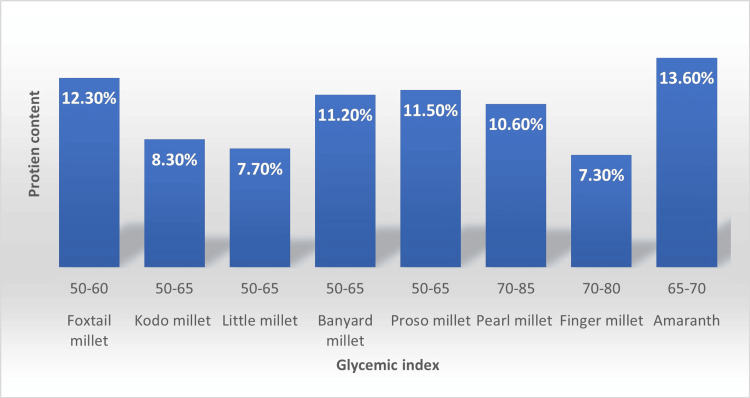
Protein composition and glycemic index of a variety of millets. Source: [28]. Image credit: Ansar Ahamed V.P.

Foods' potential to alter blood glucose levels is known as the GI. Meals with lower GI raise blood sugar levels gradually or steadily, while meals with higher GI raise blood sugar levels quickly. Numerous studies have demonstrated that the high fiber content in millet and the synthesis of resistant starch in millets delay starch hydrolysis, resulting in low GI and the potential to lower blood glucose levels [24]. In comparison to wheat and rice, millets have more dietary fiber, a non-starch polysaccharide that partially hydrolyzes the carbohydrates, protein, and fats in diets based on millet. This is because dietary fiber slows the activity of intestinal enzymes [25]. This slows the rate of absorption of mono- and disaccharides and delays the absorption of starchy polysaccharides, causing a mild glycemic response [26]. Millets produce a significant amount of resistant starch because amylose tends to retrograde starch (setback viscosity), producing resistant starch that is difficult for digestive enzymes to hydrolyze and leads to a poor glycemic response. Furthermore, the presence of fat and protein in meals slows down the rate at which the stomach empties. Consequently, this slows down the intestinal breakdown of food. Millets are known to contain higher levels of protein and fat than milled rice, which decreases their GI [27]. This results from the decreased efficiency of the small intestine's digestive process caused by many substances, including fat and protein. This results in incomplete digestion, which, in turn, causes low GI. Millet's high-protein content improves glycemic response by raising insulin sensitivity [22]. The graph in Figure 3 shows the GI and protein composition of various millet varieties found in India [28].

Importance of Diet in the Management of DM

DM is marked by an imbalance in the body's glucose homeostasis as well as abnormalities in the body's levels of proteins, lipids, and carbohydrates. In addition to causing DM, hyperglycemia also sets the stage for the emergence of numerous other disorders. The most prevalent ones include muscle exhaustion, diabetic retinopathy, atherosclerosis, demineralization-related fractures, and renal disorders [29]. DM consequences fall into two categories: acute complications and chronic problems. Two acute metabolic illnesses that can lead to mortality include diabetic ketoacidosis, resulting from unusually high blood glucose levels (hyperglycemia), and hypoglycemia, which can lead to coma. The most devastating result of diabetes is long-term vascular issues. Elevated blood glucose levels over time can lead to angiopathy and damage to blood vessels, causing a variety of problems [30]. Diabetic complications are categorized as *microvascular disease*, resulting from damage to small blood vessels, and *macrovascular disease*, which occurs due to damage to the arteries. Nephropathy, or kidney illness, neuropathy, or nerve damage, and retinopathy, or eye disease, are all considered microvascular issues. The primary macrovascular outcomes are accelerated cardiovascular disease, which causes myocardial infarction, and cerebrovascular disease, which causes strokes. There is additional cardiac dysfunction linked to diabetes, which seems to be at least somewhat independent of atherosclerosis, despite the underlying etiology being disputed [30]. Other chronic complications of DM are depression [31], dementia [32], and sexual dysfunction [33]. According to recent studies, the most common cause of illness and death is DM, and the disease's prevalence is rising daily. Globally, there has been a notable surge in the prevalence of diabetes cases as a result of factors such as population growth, aging, urbanization, rising obesity rates, and declining physical activity. According to predictions, there will be more than 350-450 million cases of diabetes worldwide during the next 10 years [34]. For individuals with diabetes, diet management is an easy and affordable strategy to improve their quality of life and provide preventive advantages. Many international diabetes groups provide recommendations for ensuring that people with diabetes get sufficient amounts of carbs, proteins, fats, fibers, and sodium in their diets to support good eating practices. The American Diabetes Association (ADA) states that proteins should make up 15% of total calories, lipids should make up 25%-35%, and carbohydrates should make up 45%-60%. Additionally, one should consume at least 14 g of fiber for every 1,000 calories, and one should restrict their daily sodium intake to no more than 2,300 mg [12]. To manage postprandial hyperglycemia and reduce body weight, the current guidelines for DM call for adopting safe, nutrient-dense diets, especially those that include low-GI starchy carbohydrates and increased dietary fiber. Research has shown that a diet high in fiber and low in GI can successfully lower plasma cholesterol and improve blood glucose regulation in individuals with DM [35].

Role of Millet-Based Diet in DM

Leading associations have established criteria that millets meet to make them the perfect food crop for individuals with diabetes. Due to the diet's high phenolic and fiber content, especially foxtail millet, it is particularly beneficial for DM [34]. A significant portion of the Indian diet is made up of millets like jowar, ragi, and bajra. For this reason, diabetologists also endorse them since they are known to encourage diabetic control measures. Because millet has a high fiber content, blood sugar levels can be lowered. Arguing that slower digestion leads to a more uniform distribution of sugar would be more appropriate [36]. By eating millet every day, a diabetic can perhaps avoid the hazardous spikes in blood sugar that lead to many issues [29]. Millets are mostly advised for patients by diabetologists due to their ability to reduce the risk of DM and cardiovascular diseases [29]. Millets have slowly digested starch, which in the intestines prolongs the process of breaking down and absorbing carbohydrates. Millet contributes to the prevention of diabetes because it releases less glucose into the blood for a longer duration than commonly consumed rice. Millets aid in body weight control, which is crucial for diabetic people. Pearl millet increases insulin sensitivity and reduces triglyceride levels in the body [12]. Table 1 shows a comparison of the elementary composition of millets with other cereals [37].

**Table 1:** A comparison of the elementary composition of millets with other cereals (per 100 g). Source: Indian Food Composition Tables and Nutritive Value of Indian foods [37].

Grains	Energy (kcal)	Protein (g)	Carbohydrate (g)	Starch (g)	Fat (g)	Dietary Fiber (g)	Minerals (g)	Ca (mg)	P (mg)
Rice	353	6.8	74.8	71	0.5	4.4	0.6	10	160
Wheat	321	11.8	64.7	56	1.5	11.2	1.5	39	306
Maize	334	11.5	64.7	59	3.6	12.2	1.5	8.9	348
Barnyard millet	307	11.6	65.5	-	5.8	-	4.7	14	121
Kodo millet	353	8.3	66.1	64	1.4	6.3	2.6	15	188
Little millet	329	8.7	65.5	56	5.3	6.3	1.7	17	220
Foxtail millet	331	12.3	60.0	-	4.3	-	3.3	31	290
Finger millet	320	7.3	66.8	62	1.3	11.1	2.7	364	283
Proso millet	341	12.5	70.0	-	1.1	-	1.9	14	206
Pearl millet	363	11.6	61.7	55	5	11.4	2.3	27	296
Sorghum	334	10.4	67.6	59	1.9	10.2	1.6	27	222

These days, millets are grains that fit into a diet quite readily. Millet should be added to the diet gradually over time, starting with a small amount at first. Millet was traditionally eaten in diets as puffed, flaked, and popped grains [38]. In recent years, millet-based noodles, vermicelli, pasta, baked goods, and sweets, particularly finger millet, have become more widely available [36]. Several tactics that enhance soil fertility, optimize cultivation practices, make optimal use of water resources, and support sustainable farming practices can all be used to increase millet production. Production of millet is important from an economic, environmental, and nutritional standpoint, and it is essential to world agriculture and food security. As part of their efforts to address food security, nutrition, and sustainable agriculture, governments around the world are putting measures and policies in place to encourage the cultivation and consumption of millet. This involves offering incentives, money for research, market support, and subsidies to promote the production of millet and its value addition. A multi-stakeholder approach is being implemented by the Indian government to commemorate the 2023 International Year of Millets (IYM). The IYM-2023 action plan is centered on methods to improve output and productivity, consumption, export, value chain strengthening, branding, and raising knowledge of health advantages, among other things. Central Ministries, State Governments, and Indian Embassies have created a yearlong action plan for monthly activities to promote the Shree Anna scheme [39]. The Department of Agriculture and Farmers Welfare (DA&FW) is executing a Sub-Mission on Nutri-Cereals under the National Food Security Mission (NFSM) across all districts of 28 states and two union territories, namely, Jammu and Kashmir and Ladakh, to boost Shree Anna's productivity and production. Farmers are given various incentives under NFSM-Nutri Cereals through the States and UTs, including cropping system-based demonstrations, certified seed of recently released varieties and hybrids, improved farm implements, tools, resource-saving machinery, water-saving devices, event or workshop organization, distribution of seed mini kits, publicity through print and electronic media, etc. Farmers are also trained during cropping season to increase their capacity [39]. In an attempt to establish India as a worldwide center for *Shree Anna*, the Indian Institute of Millets Research (IIMR), Hyderabad, has been designated as a Center of Excellence for the exchange of research, best practices, and innovations on a national and global scale [39].

## Conclusions

Millets are the future crops. They possess an energy content similar to that of regular cereals. In addition, because of their high fiber content, vitamin, mineral, and phytochemical content, as well as their ability to fight chronic illnesses, including DM, they offer greater health advantages, including antibacterial, antioxidant, and hypocholesterolemic properties. Balanced, healthful, and affordable meals can be served by including millet in a regular diet. People with diabetes can improve their general health and better regulate their blood sugar levels by using millets in their diet. Millets have been demonstrated to be an excellent blood sugar regulator due to their high fiber content and low GI. For those who have diabetes, the complex carbs in millet must break down slowly to avoid sharp rises in blood sugar. Moreover, millets have a well-rounded nutritional profile because they're an excellent source of important nutrients like minerals, vitamins, and antioxidants. These nutrients not only promote general health but also lessen the chance of problems from diabetes. Consumer awareness of nutrition is increasing, creating pressure on the food industry to develop new products with unique features that can enhance people's health. Recent research indicates that functional foods and other health-promoting materials can aid in the prevention and management of diabetes and other chronic illnesses. The review has concluded that millets have a significant impact on those with diabetes. In addition to sticking to a strict, planned, and balanced diet and medications along with engaging in regular walks or exercise, doctors, nutritionists, and patients themselves should try to incorporate millet into their meals.
